# Assessing the Short-Term Effects of Heatwaves on Mortality and Morbidity in Brisbane, Australia: Comparison of Case-Crossover and Time Series Analyses

**DOI:** 10.1371/journal.pone.0037500

**Published:** 2012-05-24

**Authors:** Shilu Tong, Xiao Yu Wang, Yuming Guo

**Affiliations:** School of Public Health and Social Work, Institute of Health and Biomedical Innovation, Queensland University of Technology, Brisbane, Queensland, Australia; Fundación para la Prevención y el Control de las Enfermedades Crónicas No Transmisibles en América Latina (FunPRECAL), Argentina

## Abstract

**Background:**

Heat-related impacts may have greater public health implications as climate change continues. It is important to appropriately characterize the relationship between heatwave and health outcomes. However, it is unclear whether a case-crossover design can be effectively used to assess the event- or episode-related health effects. This study examined the association between exposure to heatwaves and mortality and emergency hospital admissions (EHAs) from non-external causes in Brisbane, Australia, using both case-crossover and time series analyses approaches.

**Methods:**

Poisson generalised additive model (GAM) and time-stratified case-crossover analyses were used to assess the short-term impact of heatwaves on mortality and EHAs. Heatwaves exhibited a significant impact on mortality and EHAs after adjusting for air pollution, day of the week, and season.

**Results:**

For time-stratified case-crossover analysis, odds ratios of mortality and EHAs during heatwaves were 1.62 (95% confidence interval (CI): 1.36–1.94) and 1.22 (95% CI: 1.14–1.30) at lag 1, respectively. Time series GAM models gave similar results. Relative risks of mortality and EHAs ranged from 1.72 (95% CI: 1.40–2.11) to 1.81 (95% CI: 1.56–2.10) and from 1.14 (95% CI: 1.06–1.23) to 1.28 (95% CI: 1.21–1.36) at lag 1, respectively. The risk estimates gradually attenuated after the lag of one day for both case-crossover and time series analyses.

**Conclusions:**

The risk estimates from both case-crossover and time series models were consistent and comparable. This finding may have implications for future research on the assessment of event- or episode-related (e.g., heatwave) health effects.

## Introduction

Heatwaves or excessive ambient heat exposures have significant impacts on mortality and morbidity [1]–[6]. For example, during the 1995 Chicago heatwave, there were over 700 excess deaths in a single day [7]. The well-known 2003 heatwaves led to 15,000 excess deaths in France alone [8], [9], and over 70,000 deaths across Europe [10], [11]. The 2006 California heatwave resulted in an increase in morbidity which included 16,166 excess emergency department visits and 1,182 excess hospitalizations state-wide [12]. Heat-related impacts may have greater public health implications as climate change continues. It is important to appropriately characterize the relationship between heatwaves and health outcomes.

Two common epidemiologic methods have been frequently used to assess the heat-related health effects. Time series analysis has been used to investigate the health impact of time varying environmental exposures (eg, air pollution and temperature) for many years [13], [14]. Recently, a case-crossover design (introduced by MaClure in 1991) has been increasingly used to examine an association between a transient exposure (eg, temperature or air pollution) and acute health outcomes [15], [16]. This design controls for time-invariant confounders by study design itself [17]. Therefore, it has some advantages compared with commonly-used time series analysis. However, some methodological issues in the use of case-crossover analysis have attracted much research attention. For example, unidirectional case-crossover design was initially applied and the referent period was designated by specific time period(s) before the case period [18]. Recently, ambidirectional and time-stratified case-crossover analyses have been assumed as ideal approaches because unidirectional design has often produced biased results [18]–[20]. The previous research mainly focused on the risk assessment of time-varying exposures (eg, air pollution and temperature) using relatively long time series datasets. However, little information is available on whether these findings are applicable to the assessment of event- or episode-related (eg, heatwave) health effects. Since time series and case-crossover methods are often viewed as two competing analytical approaches, this study examined whether these methods produced equivalent risk estimates in the assessment of the health effects of heatwaves in Brisbane, Australia.

## Materials and Methods

### Data collection

Brisbane, Australia's third largest city, is located in the south-east corner of the Queensland state (27°29′S, 153°8′E) and has a sub-tropical climate. The population increased from 896,649 on 30 June 2001 to 991 260 on 30 June 2006. 18% of the residents in Brisbane were aged 0–14, while 11% of them were aged 65+[21].

We obtained emergency hospital admissions (EHAs) data during 1st January 1996 to 31st December 2005, and mortality data during 1st January 1996 to 30th November 2004. Daily data on mortality and EHAs were provided by the Office of Economic and Statistical Research of the Queensland Treasury and the Health Information Centre of Queensland Health, respectively. Non-external causes (NEC) mortality and EHAs were categorised according to the International Classification of Diseases (revisions 9 and 10) (ICD 9, <800; and all ICD 10 codes excluding S00–U99 for external causes).

Daily data on maximum temperature and relative humidity data were obtained from the Australian Bureau of Meteorology during January 1996 to December 2005. The daily average values of climatic variables were calculated from five monitoring stations. We retrieved daily air pollution data from the Queensland Department of Environment and Resource Management (formerly Queensland Environmental Protection Agency), including ambient 24-hour average concentrations of particulate matter with diameter less than 10 µm (PM_10_), daily maximum 1-hour average nitrogen dioxide (NO_2_) and ozone (O_3_). Daily air pollution concentrations were averaged from seventeen monitoring stations in Brisbane. When data were missing for a particular monitoring station on a given day, the data recorded from other monitoring stations were used to calculate the daily average values.

### Data analysis

According to the local heatwave definition (ie, daily maximum temperature higher than 37°C for two or more consecutive days) developed in the previous research [22], [23], three heatwaves occurred (20 and 21 January 2000; 24 to 26 December 2001; 21 and 22 February 2004) during the whole study period. We examined the short-term effects of heatwaves on mortality and EHAs within three different periods, 84 days (28 days as a strata length for time-stratified case-crossover analysis), summer season (from December to February) and whole study period (1996–2005). Spearman's correlation coefficients were used to evaluate the interrelations between air pollutants and climate variables in these three periods.

In this study, both time-series and case–crossover analyses were used to examine the heatwave effects on NEC mortality and EHAs. Poisson generalised additive model (GAM) was used to perform time series analyses on three different periods. We also used time-stratified case–crossover with a stratum length of 28 days, and matched case-control days using day of the week (one case day was matched with three control days). Because only three heatwaves were defined during the whole study period, **t**hree strata (84 days) were used for the case–crossover analysis. Heatwave days were categorised as 1, while non-heatwave days were categorised as 0. We adjusted for humidity and air pollutants (PM_10_, NO_2_ and O_3_) in these models. Additionally, lagged effects (ie, lag 0 to lag 5 and moving average of lags 0–5) were assessed using the same methods. When lagged effects of heatwaves on NEC mortality and EHAs were assessed, the same lagged effects of humidity and air pollution were controlled for. Relative risks (RRs) for time series analysis, odds ratios (ORs) for time-stratified case–crossover analysis, and 95% confidence intervals (CIs) were calculated in each model. R software (version 2.12.2) and the “mgcv” package were applied to fit the time series GAM and case–crossover analyses. The R codes are provided in Information S1 using an example dataset (Information S2), and results of estimation using different degree of freedom were presented in Figure S1.

## Results

There were a total of 51,233 deaths and 488,005 EHAs recorded in Brisbane during the whole study period. Table 1 shows summary statistics of climatic variables, air pollutants, mortality and EHAs for three different periods, as well as the total number of different health outcomes used in each model. There was some variation in these variables during three periods. The mean concentrations of PM_10_, NO_2_ and O_3_ differed slightly across the different periods. The daily average deaths (17) and EHAs (142) during 84 days were higher than those during summer days (15 deaths and 128 EHAs) or whole study period (16 deaths and 134 EHAs).

**Table 1:** Summaries of daily weather, air pollutants and health outcomes in Brisbane, Australia.

	84 days			Summer			1996–2005		
	Mean	SD	Range	Mean	SD	Range	Mean	SD	Range
Tmax^a^ (°C)	30.9	3.8	21.0 to 41.5	30.0	2.6	21.0 to 41.5	26.3	3.9	12.6 to 41.5
Humidity (%)	70.8	8.1	44.9 to 90.2	71.7	8.1	39.6 to 96.9	71.1	10.3	24.6 to 96.9
PM_10_ (µg/m^3^)	21.1	8.9	7.2 to 43.7	17.8	6.7	6.5 to 78.6	17.7	7.6	2.5 to 151.6
NO_2_ (ppb)	13.1	4.2	5.2 to 26.8	12.1	4.0	3.6 to 30.8	18.0	6.7	3.3 to 46.3
O_3_ (ppb)	35.4	12.3	13.9 to 67.8	31.7	12.1	7.7 to 88.2	31.8	9.8	7.1 to 88.2
Deaths	17	6	5 to 4	15	4	5 to 42	16	4	5 to 42
			(1,400)^c^			(11,432)^c^			(51,233)^c^
EHAs^b^	142	21	101 to 202	128	21	71 to 202	134	22	71 to 212
			(11,962)^c^			(115,406)^c^			(488,005)^c^

a maximum temperature.

b emergency hospital admissions.

c the number of outcomes.

Table 2 shows that maximum temperatures positively correlated with most air pollutants in all periods except for NO_2_ during the whole study period. Moderate to high correlations were observed for temperature and PM_10_, NO_2_ and ozone. However, humidity inversely correlated with most air pollutants in different periods, although most of these correlations were week. There were significant associations between air pollutants.

**Table 2:** Spearman correlation between climatic variables and air pollutants in Brisbane, Australia.

	Humidity	PM_10_	NO_2_	O_3_
84 days				
Tmax^a^ (°C)	−0.18	0.61**	0.72**	0.84**
Humidity (%)		−0.40**	−0.08	−0.10
PM_10_ (µg/m^3^)			0.40**	0.62**
NO_2_ (ppb)				0.68**
Summer				
Tmax^a^ (°C)	−0.06	0.35**	0.24**	0.65**
Humidity (%)		−0.31**	0.01	−0.09*
PM_10_ (µg/m^3^)			0.33**	0.46**
NO_2_ (ppb)				0.51**
1996–2005				
Tmax^a^ (°C)	−0.06**	0.25**	−0.47**	0.35**
Humidity (%)		−0.26**	−.0.06**	−0.20**
PM_10_ (µg/m^3^)			0.30**	0.47**
NO_2_ (ppb)				0.28**

a Tmax = maximum temperature.

* *P*<0.05.

** *P*<0.01.

In time-stratified case-crossover analysis we used 28 days as a stratum length. Since three heatwaves occurred during the study period, therefore 84 days (ie, three strata: 3×28 days) were used in data analysis. Table 3 shows the estimated odds ratios (ORs) and relative risks (RRs) for NEC mortality and EHAs during heatwave days compared with non-heatwave days during these 84 days. There was a broadly consistent and statistically significant increase in ORs or RRs for both mortality and EHAs during heatwaves.

**Table 3:** Odds ratios (ORs) and Relative risks (RRs) of mortality and EHAs during heatwaves in Brisbane (84 days).

	OR/RR (95% CI)			
HW effect	Model I^a^	Model II^b^	Model III^c^	Model IV^d^
Deaths				
Case-crossover				
Lag 0	1.53 (1.27, 1.85)	1.47 (1.22, 1.78)	1.52 (1.25, 1.83)	1.51 (1.25, 1.83)
Lag 1	1.67 (1.40, 2.00)	1.63 (1.36, 1.95)	1.65 (1.38, 1.97)	1.64 (1.38, 1.96)
Lag 2	1.39 (1.16, 1.67)	1.37 (1.14, 1.65)	1.39 (1.15, 1.67)	1.38 (1.15, 1.66)
Lag 3	1.15 (0.94, 1.42)	1.16 (0.94. 1.42)	1.15 (0.94, 1.41)	1.14 (0.93, 1.41)
Lag 4	1.18 (0.96, 1.46)	1.17 (0.95, 1.45)	1.17 (0.95, 1.45)	1.18 (0.95, 1.45)
Lag 5	1.15 (0.92, 1.44)	1.19 (0.95, 1.49)	1.16 (0.93, 1.45)	1.16 (0.93, 1.45)
6-day average	1.36 (1.12, 1.65)	1.37 (1.12, 1.66)	1.36 (1.11, 1.65)	1.35 (1.11, 1.64)
Time-series				
Lag 0	1.44 (1.22, 1.70)	1.26 (1.03, 1.54)	1.48 (1.23, 1.79)	1.25 (1.02, 1.55)
Lag 1	1.72 (1.48, 2.01)	1.83 (1.51, 2.22)	1.99 (1.67, 2.38)	1.70 (1.39. 2.08)
Lag 2	1.47 (1.25, 1.73)	1.50 (1.22, 1.83)	1.66 (1.38, 2.00)	1.45 (1.18, 1.79)
Lag 3	1.10 (0.92, 1.33)	1.14 (0.92, 1.42)	1.21 (0.98, 1.48)	1.07 (0.85, 1.35)
Lag 4	1.04 (0.87, 1.26)	1.05 (0.84, 1.31)	1.12 (0.91, 1.39)	1.09 (0.86, 1.37)
Lag 5	0.94 (0.77, 1.14)	1.05 (0.83, 1.33)	1.02 (0.82, 1.27)	1.13 (0.89, 1.44)
6-day average	1.28 (1.08, 1.52)	1.29 (1.07, 1.56)	1.29 (1.078, 1.564	1.29 (1.07, 1.55)
EHAs				
Case-crossover				
Lag 0	1.17 (1.10, 1.26)	1.16 (1.08, 1.24)	1.16 (1.09, 1.25)	1.17 (1.10, 1.26)
Lag 1	1.23 (1.15, 1.31)	1.22 (1.14, 1.30)	1.23 (1.15, 1.32)	1.23 (1.15, 1.32)
Lag 2	1.17 (1.10, 1.26)	1.17 (1.09, 1.25)	1.18 (1.10, 1.26)	1.18 (1.10, 1.26)
Lag 3	1.13 (1.05, 1.21)	1.13 (1.05, 1.21)	1.13 (1.05, 1.21)	1.13 (1.05, 1.21)
Lag 4	1.11 (1.03, 1.19)	1.10 (1.03, 1.18)	1.11 (1.03, 1.19)	1.11 (1.03, 1.19)
Lag 5	1.04 (0.97, 1.12)	1.04 (0.97, 1.11)	1.04 (0.97, 1.12)	1.04 (0.97, 1.11)
6-day average	1.14 (1.07, 1.23)	1.15 (1.07, 1.23)	1.14 (1.07, 1.23)	1.14 (1.07, 1.23)
Time-series				
Lag 0	1.14 (1.07, 1.21)	1.08 (1.01, 1.17)	1.12 (1.04, 1.20)	1.09 (1.01, 1.17)
Lag 1	1.20 (1.13, 1.28)	1.15 (1.07, 1.24)	1.19 (1.11, 1.27)	1.13 (1.05, 1.22)
Lag 2	1.16 (1.10, 1.24)	1.13 (1.05, 1.21)	1.18 (1.10, 1.26)	1.11 (1.03, 1.20)
Lag 3	1.09 (1.03, 1.16)	1.06 (0.98, 1.14)	1.11 (1.03, 1.19)	1.03 (0.96, 1.12)
Lag 4	1.07 (1.01, 1.14)	1.03 (0.96, 1.11)	1.08 (1.01, 1.16)	1.01 (0.94, 1.10)
Lag 5	1.03 (0.97, 1.10)	1.01 (0.94, 1.09)	1.04 (0.97, 1.12)	0.99 (0.92, 1.07)
6-day average	1.12 (1.05, 1.19)	1.04 (0.97, 1.11)	1.08 (1.01, 1.15)	1.05 (0.99, 1.12)

a unadjusted.

b adjusted for humidity and O_3_.

c adjusted for humidity and PM_10_.

d adjusted for humidity and NO_2_.

**Figure 1:**
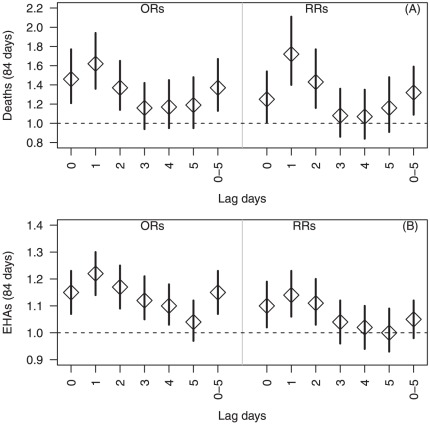
Odds ratios and Relative risks of daily mortality (1A) and emergency hospital admissions (1B) during heatwaves (84 days), using both case crossover and time series analyses.

Figure 1 shows the similar results as Table 3 when both methods was used to adjust for humidity, PM_10_, NO_2_ and O_3_. ORs of mortality and morbidity during heatwaves were 1.62 (95% CI: 1.36–1.94) (Figure 1A) and 1.22 (95% CI: 1.14–1.30) (Figure 1B) at lag 1, respectively. RRs of mortality and morbidity during heatwaves were 1.72 (95% CI: 1.40–2.11) (Figure 1A) and 1.14 (95% CI: 1.06–1.23) (Figure 1B) at lag 1, respectively. The risk estimates also generally alleviated after lag 1 for both mortality and EHAs.

Table 4 shows the delayed effects of heatwaves on NEC mortality and EHAs for summer season and whole study period using time series models to adjust for different sets of confounders. There were also broadly consistent increased risks for both mortality and EHAs during heatwaves across different periods. The highest risk estimates were observed at lag 1 for both mortality and EHAs after adjusting for confounders.

**Table 4:** Relative risks (RRs) of daily mortality and EHAs during heatwaves in Brisbane (summer season and whole study period), using time series analysis.

	OR/RR (95% CI)			
HW effect	Model I^a^	Model II^b^	Model III^c^	Model IV^d^
Deaths				
Summer				
Lag 0	1.59 (1.36, 1.86)	1.49 (1.27, 1.75)	1.53 (1.31, 1.80)	1.54 (1.32, 1.81)
Lag 1	1.87 (1.62, 2.16)	1.80 (1.55, 2.08)	1.85 (1.60, 2.14)	1.86 (1.60, 2.15)
Lag 2	1.64 (1.41,1.91)	1.60 (1.36, 1.87)	1.63 (1.39, 1.90)	1.63 (1.39, 1.91)
Lag 3	1.24 (1.04, 1.48)	1.22 (1.02, 1.46)	1.23 (1.03, 1.47)	1.24 (1.04, 1.48)
Lag 4	1.17 (0.98, 1.40)	1.15 (0.96, 1.38)	1.17 (0.98, 1.41)	1.18 (0.98, 1.42)
Lag 5	1.05 (0.87, 1.27)	1.07 (0.88, 1.30)	1.06 (0.88, 1.29)	1.09 (0.90, 1.32)
6-day average	1.42 (1.21, 1.67)	1.39 (1.18, 1.65)	1.40 (1.19, 1.65)	1.42 (1.21, 1.68)
1996-2005				
Lag 0	1.47 (1.26, 1.72)	1.44 (1.23, 1.68)	1.45 (1.25, 1.70)	1.49 (1.27, 1.73)
Lag 1	1.73 (1.50, 1.99)	1.71 (1.48, 1.98)	1.71 (1.48, 1.97)	1.73 (1.50, 2.00)
Lag 2	1.52 (1.30, 1.77)	1.53 (1.31, 1.78)	1.51 (1.30, 1.76)	1.52 (1.31, 1.77)
Lag 3	1.15 (0.97, 1.37)	1.17 (0.98, 1.39)	1.15 (0.96, 1.37)	1.16 (0.97, 1.38)
Lag 4	1.09 (0.91, 1.30)	1.10 (0.92, 1.32)	1.08 (0.91, 1.30)	1.09 (0.91, 1.30)
Lag 5	0.98 (0.81, 1.18)	1.02 (0.84, 1.23)	0.99 (0.82, 1.19)	0.99 (0.82, 1.19)
6-day average	1.32 1.12, 1.55)	1.37 (1.16, 1.61)	1.33 (1.13, 1.56)	1.39 (1.18, 1.64)
EHAS				
Summer				
Lag 0	1.26 (1.19, 1.34)	1.21 (1.14, 1.29)	1.20 (1.13, 1.27)	1.23 (1.15, 1.30)
Lag 1	1.32 (1.25, 1.40)	1.28 (1.21, 1.36)	1.27 (1.19, 1.34)	1.30 (1.22, 1.38)
Lag 2	1.29 (1.22, 1.37)	1.26 (1.18, 1.33)	1.24 (1.17, 1.31)	1.27 (1.19, 1.34)
Lag 3	1.21 (1.14, 1.29)	1.19 (1.12, 1.26)	1.17 (1.10, 1.24)	1.20 (1.13, 1.28)
Lag 4	1.18 (1.11, 1.26)	1.15 (1.09, 1.23)	1.14 (1.07, 1.21)	1.18 (1.11, 1.25)
Lag 5	1.14 (1.07, 1.21)	1.12 (1.05, 1.19)	1.10 (1.03, 1.17)	1.13 (1.07, 1.21)
6-day average	1.23 (1.16, 1.31)	1.22 (1.15, 1.29)	1.19 (1.12, 1.26)	1.23 (1.16, 1.30)
1996–2005				
Lag 0	1.20 (1.13, 1.27)	1.20 (1.13, 1.27)	1.18 (1.11, 1.25)	1.20 (1.13, 1.27)
Lag 1	1.26 (1.19, 1.34)	1.27 (1.20, 1.34)	1.25 (1.18, 1.32)	1.26 (1.19, 1.34)
Lag 2	1.23 (1.17, 1.31)	1.24 (1.17, 1.32)	1.22 (1.15, 1.29)	1.23 (1.16, 1.30)
Lag 3	1.16 (1.09, 1.23)	1.17 (1.10, 1.25)	1.15 (1.08, 1.22)	1.15 (1.09, 1.22)
Lag 4	1.13 (1.07, 1.20)	1.15 (1.08, 1.22)	1.12 (1.06, 1.19)	1.13 (1.06, 1.20)
Lag 5	1.09 (1.02, 1.16)	1.10 (1.04, 1.17)	1.08 (1.01, 1.15)	1.08 (1.02, 1.15)
6-day average	1.18 (1.11, 1.25)	1.22 (1.15, 1.29)	1.17 (1.11, 1.25)	1.20 (1.13, 1.27)

a unadjusted.

b adjusted for humidity and O_3_.

c adjusted for humidity and PM_10_.

d adjusted for humidity and NO_2_.

**Figure 2:**
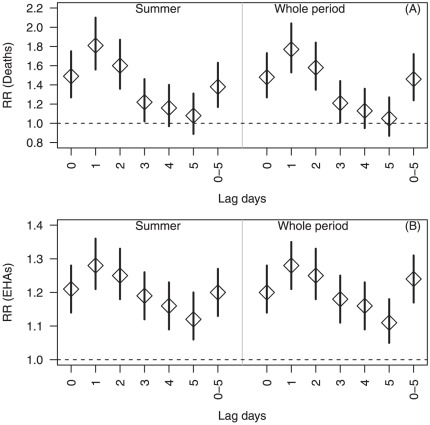
Relative risks of daily mortality (2A) and emergency hospital admissions (2B) during heatwaves (summer season and whole study period), using time series analyses.

Figure 2 reveals the similar results as Table 4 when time series GAM models were used with different periods to adjust for humidity, PM_10_, NO_2_ and O_3_. The results show that RRs of mortality and EHAs ranged from 1.77 (95% CI: 1.53–2.04) to 1.81 (95% CI: 1.56–2.10) in Figure 2A and 1.28 (95% CI: 1.21–1.36) (Figure 2B) at lag 1, respectively. The risk estimates generally attenuated after the lag of one day for both mortality and EHAs.

## Discussion

An increase in the frequency, duration and intensity of heatwaves is one of the most certain impacts of global climate change [24], and therefore, it is important to characterise the heat-related health risks. In this study, we found consistent and significant risks of NEC deaths and EHAs during heatwaves using both time series and case-crossover methods. For time series analyses, a similar pattern of heatwave-related risks was observed even though different periods of data were used. In general, the effect estimates using time series analyses were quite comparable to those estimated by the case-crossover method. We also found that heatwaves had acute effects on mortality and EHAs, and the highest impact usually occurred at lag 1 in Brisbane – a subtropical city in Australia.

In a recent study of the relationship between temperature and mortality among the elderly, the results from a case-crossover study design using three different approaches for the selection of referent periods were compared with those from the time-series analysis [19]. Consistent results were found by using either the time-series or time-stratified case-crossover analysis. Our findings support and extend the previous study, demonstrating that time-stratified case-crossover design can also be used to estimate the event or episode-related (e.g., heatwave) health effects, and both time series and time-stratified case-crossover methods produced robust and comparable results.

Previous studies reported that increases in mortality and morbidity usually occurred within a short time frame after a heatwave [5], [25]–[28]. For example, in the 2003 French heatwave, relative risk for mortality increased rapidly in most cities during a 4-day period from 10 to 13 August [9]. Similarly, in the Chicago heatwave, there was the highest dose–response relationship for lags of 1 and 2 days of high minimum or maximum temperatures before all-cause mortality occurred, with an RR of 1.20 (95% CI: 1.15–1.27) for high temperature on lag day 1 and an indication of a cumulative effect (RR = 1.40; 95% CI:1.30–1.51) when temperatures stayed high both on lag days 1 and 2 [15]. In our study, we also found that exposure to heatwaves at the same day, lag 1 and lags 0–5 was strongly associated with risks of mortality and EHAs. The strongest heatwave effects appeared at lag 1 and lagged effects were gradually decreased after lag 1 for both EHAs and mortality (Figures 1 and 2).

Previous studies including our own research show an immediate impact of high temperature on the patients with cardiovascular diseases [29], suggesting that cardiovascular deaths tend to happen quickly during hot days because their ability to cope with heat is already compromised. Several pathophysiological mechanisms may explain this. Firstly, heat stress can reduce cerebral blood velocity and markedly impair orthostatic tolerance in humans [23], [30]. Secondly, water loss and reduced plasma volume during hot days may facilitate the release of platelets into circulation and increase red and white cell counts, blood viscosity, and plasma cholesterol levels, which may be connected to the increased mortality from arterial thrombosis in hot weather [31]. Finally, results from a mice study indicate that heat stress will stimulate cells of living organisms to generate heat shock proteins which may cause systematic damages in the body [32].

This study has three main strengths: (1) it is the first study to compare time series and case crossover analyses in the examination of heatwave effects on mortality and EHAs. Time-stratified case-crossover design is comparable to time series analysis in estimating event or episode-related health risks; (2) sophisticated statistical methods were used to assess lagged and cumulative average effects of heatwaves on both mortality and EHAs after adjustment for confounding factors; and (3) the datasets used is this study are quite comprehensive, with no missing values.

Several limitations of this study must also be acknowledged. We only focused on one city, so the results might not be generalisable to other areas. However, the approaches applied in this study can be used in further research in other areas. We only compared time series and case crossover analyses using the data on NEC mortality and EHAs. We did not use cause-specific mortality and morbidity because the number for each category was much smaller. Additionally, there might be exposure misclassification, as we used exposure data from fixed monitors rather than individual exposure data.

In conclusion, the results of this study demonstrate that both time-stratified case-crossover and time series analyses produced a similar pattern of the relationship between heatwave and health outcomes. This finding may have implications for future studies of event or episode-related health effects.

## Supporting Information

Figure S1 **The coefficients of temperature effects on mortality using different degrees of freedom for day of the year.**
(TIFF)

Information S1 **Supplemental Material.**
(DOCX)

Information S2 **Example dataset.**
(CSV)
