# Supplementary figures and images for: Assessing the Short-Term Effects of Heatwaves on Mortality and Morbidity in Brisbane, Australia: Comparison of Case-Crossover and Time Series Analyses
Source: PLoS One. 2012 May 24;7(5):e37500. doi: 10.1371/journal.pone.0037500 (PMC3360052; doi:10.1371/journal.pone.0037500)

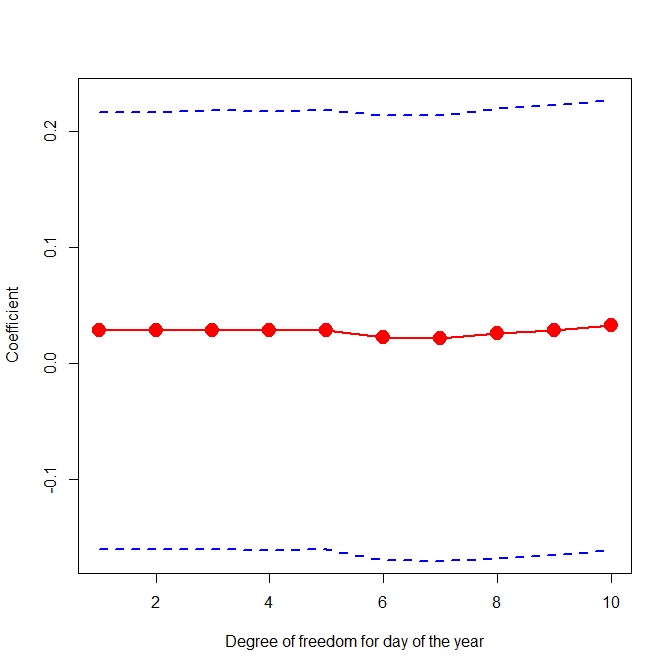

Supplement: Figure S1 — The coefficients of temperature effects on mortality using different degrees of freedom for day of the year. (TIFF) [file pone.0037500.s001.tiff]
