# Supplementary material for: Assessing the Short-Term Effects of Heatwaves on Mortality and Morbidity in Brisbane, Australia: Comparison of Case-Crossover and Time Series Analyses
Source: PLoS One. 2012 May 24;7(5):e37500. doi: 10.1371/journal.pone.0037500 (PMC3360052; doi:10.1371/journal.pone.0037500)
Supplement: Information S1 — Supplemental Material. (DOCX) [file pone.0037500.s002.docx]

**Supplemental Material**

**Assessing the short-term effects of heatwaves on mortality and morbidity in Brisbane, Australia: Comparison of case-crossover and time series analyses**

Shilu Tong,* Xiao Yu Wang, Yuming Guo

School of Public Health and Social Work, Institute of Health and Biomedical Innovation, Queensland University of Technology, Kelvin Grove, QLD 4059, Australia

**Supplemental Material, R code**

Since our data from Brisbane is not publicly available, we created a dataset as an example (example.csv).

1. **Load package and prepare dataset:**

library(mgcv) ### get package

data<-read.csv("example.csv",head=T) ### get data

data$stratadow<-paste(data$strata28,data$dow,sep="") ### create strata and day of week

1. **Fit case-crossover design**

#Unadjusted

cc1<-gam(death ~ hw+as.factor(stratadow), family=poisson, data=data)

#Adjust humidity

cc2<-gam(death ~ hw+as.factor(stratadow)+rhum, family=poisson, data=data)

#Adjust PM10

cc3<-gam(death ~ hw+as.factor(stratadow)+pm10, family=poisson, data=data)

#Adjust PM10 and humidity

cc4<-gam(death ~ hw+as.factor(stratadow)+pm10+rhum, family=poisson, data=data)

1. **Fit time series design**

#Select degree of freedom for day of the year using unadjusted model

coef<-rep("NA",10)

coef.l<-rep("NA",10)

coef.h<-rep("NA",10)

for (i in 1:10){

ts<-gam(death ~ hw+as.factor(dow)+s(doy,k=i+1,fx=T), family=poisson, data=data)

summary(ts)

coef[i]<-summary(ts)$p.coeff[2]

coef.l[i]<-summary(ts)$p.coeff[2]-1.96*summary(ts)$se[2]

coef.h[i]<-summary(ts)$p.coeff[2]+1.96*summary(ts)$se[2]

}

plot (1:10, coef, ylim=c(-0.165,0.23),pch=19,cex=2,col=2,

xlab="Degree of freedom for day of the year", ylab="Coefficient")

lines(1:10,coef,col=2,lwd=2)

lines(1:10,coef.l,col=4,lwd=2,lty=2)

lines(1:10,coef.h,col=4,lwd=2,lty=2)

Information SFigure 1 shows that the estimates are similar using different degree of freedom, so we used 3 degrees of freedom for day of the year in this example study.

#Unadjusted model

ts1<-gam(death ~ hw+as.factor(dow)+s(doy,k=4,fx=T), family=poisson, data=data)

#Adjust humidity

ts2<-gam(death ~ hw+as.factor(dow)+s(doy,k=4,fx=T)+rhum, family=poisson, data=data)

#Adjust PM10

ts3<-gam(death ~ hw+as.factor(dow)+s(doy,k=4,fx=T)+pm10, family=poisson, data=data)

#Adjust PM10+humidity

ts4<-gam(death ~ hw+as.factor(dow)+s(doy,k=4,fx=T)+pm10+rhum, family=poisson, data=data)
